# Reviewing the Possible Rare Reasons for Normal Parathyroid Hormone, Vitamin D and Serum Calcium Levels in a Patient With Severe Osteoporosis and Radiologic Parathyroid Adenoma: A Case Report

**DOI:** 10.7759/cureus.73901

**Published:** 2024-11-18

**Authors:** Alamin Alkundi, Rabiu Momoh

**Affiliations:** 1 Diabetes and Endocrinology, East Kent Hospitals University NHS Foundation Trust, Kent, GBR; 2 Critical Care, Medway Maritime Hospital, Gillingham, GBR

**Keywords:** calcium homeostasis, fragility fracture, osteoporosis, parathyroid adenoma, parathyroidectomy, parathyroid hormone, parathyroid hormone-related peptide (pthrp), romosumab, sestamibi scan, teriparatide

## Abstract

Literature evidence describing a seeming de novo occurrence of severe osteoporosis accompanied by the presence of parathyroid adenoma with normal serum parathormone level (PTH), normal serum vitamin D, and serum calcium levels is rare; hence, this case report. In the absence of raised parathormone levels and the demonstration of the presence of parathyroid adenoma, the hypothesis that the authors were left with was that could certain forms of parathyroid adenoma express factors or active substances with severe osteoclastic activity. Or, could certain expressed PTH molecules in parathyroid adenoma scenarios prove difficult to assay using conventional study methods? We have reviewed the literature in a bid to provide answers to these possible uncommon scenarios. We present the case of the above occurrence in a 65-year-old female patient with no genetic evidence of familial hypocalciuric hypercalcemia or familial hyperparathyroidism and with a normal renal function test.

## Introduction

Parathyroid adenomas are benign encapsulated parathyroid gland tumors and are composed of chief cells, oncocytes, transitional oncocytes, or mixtures of these cell types. Parathyroid adenoma has an incidence rate of 1:1000, and women are more often affected than men. They are largely known to be accompanied by an increased expression of serum parathormone level (PTH) and hypercalcemia and are often associated clinically with osteoporosis, a condition of increased bone osteoclastic activity more than bone formation. The peak incidence of this condition is from the fourth to the sixth decades of life. The patients may also present with multisystemic symptoms and signs of hypercalcemia [1]. Parathyroidectomy is often recommended for the treatment of parathyroid adenoma that is associated with osteoporosis or renal failure [2]. Parathyroid adenomas can occur in isolation or as a component of a larger syndrome (e.g., multiple endocrine neoplasia (MEN 1 syndrome)) [1].

The rare occurrence of normocalcemic primary hyperparathyroidism is gaining attention in the endocrinology literature. The true incidence of this condition is largely unknown, but an estimated prevalence of 0.4% to 0.6% has been observed among asymptomatic patients [3]. We present an even rarer case involving a female patient in her mid-sixties who had a 12-year history of recurrent fractures. She was found to have radiologic evidence of a parathyroid adenoma, yet her serum calcium, vitamin D levels, renal function, and PTH levels were mostly found within normal reference ranges.

## Case presentation

**Figure 1:**
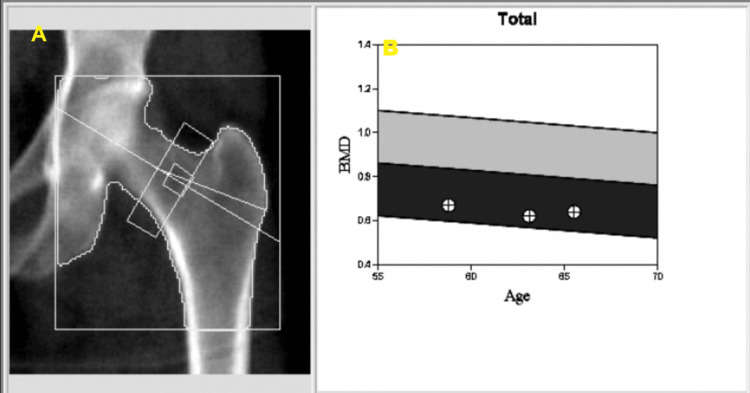
Evidence of osteoporosis affecting the left hip joint Panel A: The latest dual-energy X-ray absorptiometry (DEXA) scan study assessing the patient's left hip joint revealed evidence of osteoporosis. Left Hip bone mineral density (BMD) assessed as 0.638 g/cm2; T-score:-2.5; Z-score:-1.2; Classified as: Osteoporosis Panel B: The serial trend of low BMD results assessed in that joint is also presented as a graph.

**Figure 2:**
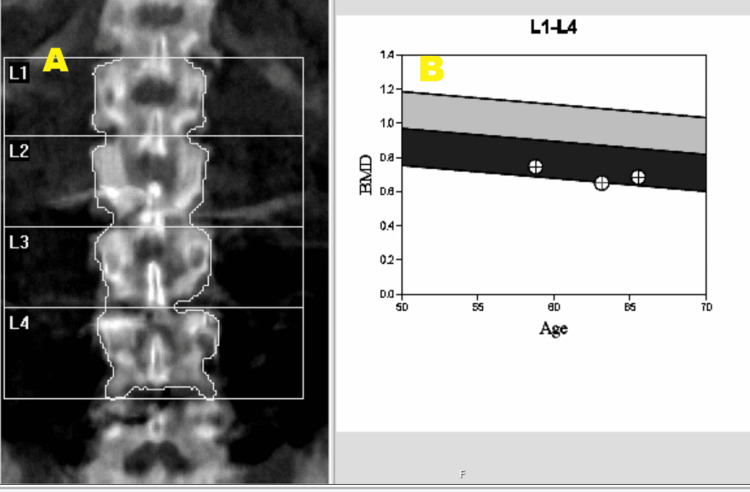
Evidence of osteoporosis affecting the lumbar vertebrae (L1-L4) Panel A: Latest DEXA scan study on patient assessing the lumbar vertebrae (L1-L4). BMD: 0.684 g/cm2; T-score:-3.3; Z-score:-1.5; Classified as: Osteoporosis Panel B: A graph depicts the serial trend of BMD results for the L1-L4 lumbar vertebrae. BMD: Bone mineral density; DEXA: Dual-energy X-ray absorptiometry

We present the case of a 65-year-old female patient who was evaluated at an endocrinology clinic. She had a history of multiple fractures over 12 years (Table 1) and was diagnosed with osteoporosis seven years before this report (Figure 1-2). Additionally, her past medical history includes non-debilitating relapsing-remitting multiple sclerosis (MS), which was diagnosed 19 years before the index date, for which she remained fully independent and active. She was on a disease-modifying therapy with Copaxone (glatiramer acetate injection) for her MS for the first twelve years since the MS was diagnosed. Before this clinic visit, her regular medications included dimethyl fumarate (Tecfidera) (which she had been using for seven years till the date of this publication), hormone replacement therapy (oestradiol with norethisterone), and vitamin D supplements.

**Table 1:** Fracture history in the patient

Date	Site	Fracture type	Comment
12 years prior to the case report	Lower leg	High impact	Sustained while skiing
7 years prior to the case report	Wrist	Fragility	Right wrist
3 years prior to the case report	Thoracic vertebrae	Fragility	MRI spine study done revealed height loss at multiple levels, mainly T12
Year of the case report	Thoracic vertebrae	Fragility	Multiple minor compressions

The patient chose not to be treated with bisphosphonate over 6 years in the light of the risk of osteonecrosis of the jaw with antiresorptive agent use due to her ongoing management for dormant dental abscesses with a dental center that has spanned over 20 years. At a visit to an osteoporosis clinic a year prior, she was considered for treatment with hormone replacement therapy at that visit. She was again seen at the osteoporosis clinic within the year of this publication, and the potential for treatment with teriparatide or romosozumab (both anabolic bone-forming agents) was considered. The use of teriparatide was reviewed as contra-indicated because of a recent assessment of parathyroid adenoma in the patient.

**Figure 3:**
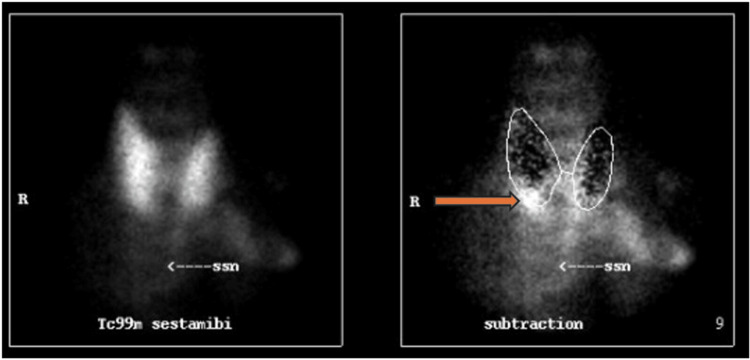
Focal sestamibi uptake along the lower pole of the right thyroid lobe is identified with the arrow

**Figure 4:**
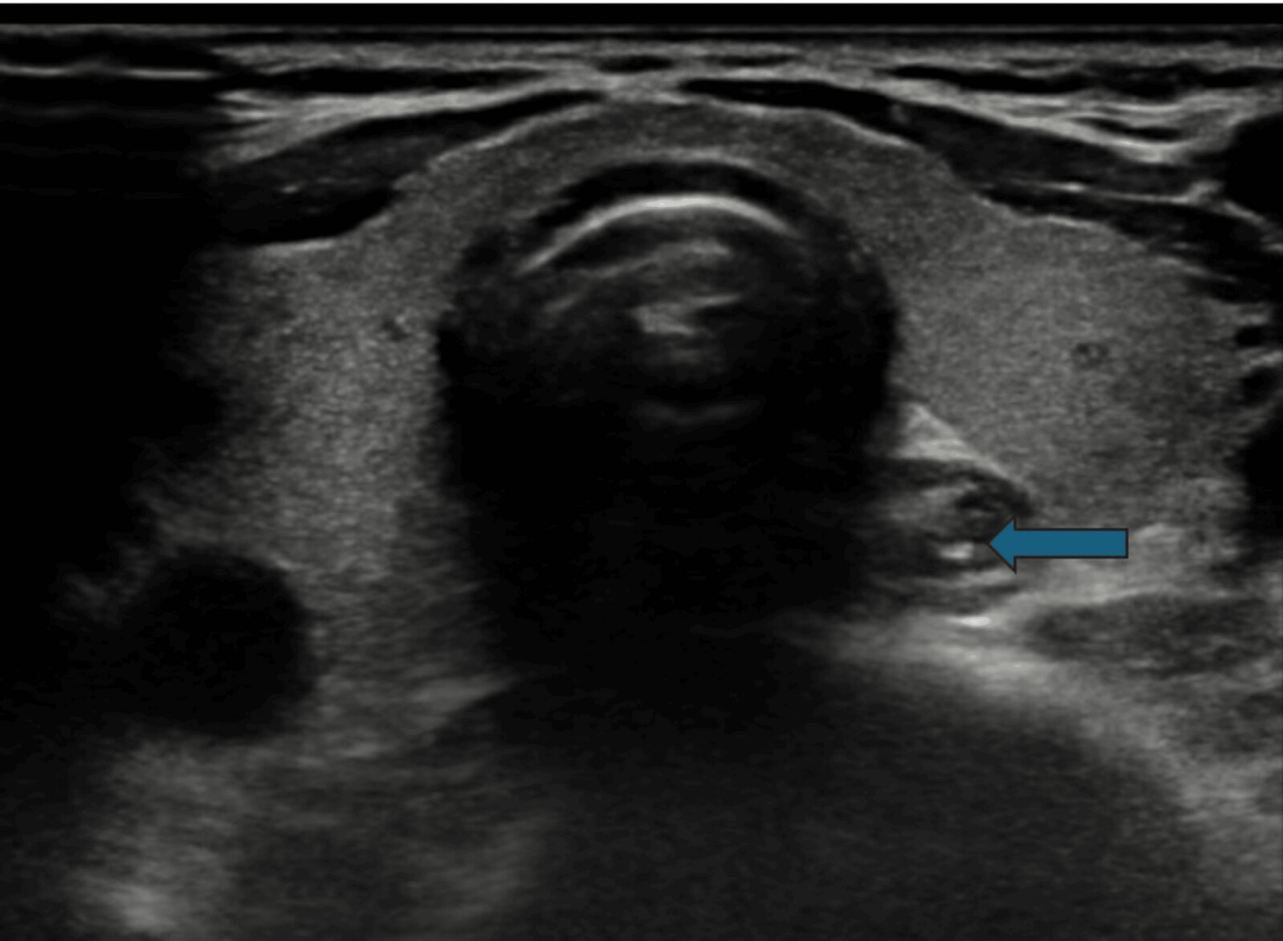
Nodular area postero-inferior and lateral to the lower pole of the right lobe of the thyroid depicting the radiologic parathyroid adenoma

The patient was diagnosed with radiologic right parathyroid adenoma on a sestamibi parathyroid nuclear scan study done 11 months prior to this publication. Focal Sestamibi uptake along the lower pole of the right thyroid lobe was demonstrated (Figure 3). She was, however, noted to have normal serum calcium studies and was vitamin D replete. Ultrasound neck study also revealed a hypoechoic nodular area measuring 10 mm posterior inferior and lateral to the lower pole of the right lobe of the thyroid with a homogeneous appearance and no vascularity or calcification possibly representing the parathyroid nodule noted on the sestamibi scan (Figure 4).

There were multiple nodules in the thyroid gland, mainly involving the right lobe. Most of these nodules are cystic, with the largest measuring 5 mm, and should represent U one category features. There are also a few small ill-defined hypoechoic areas in the right lobe. The rest of the thyroid gland was unremarkable, with homogeneous background parenchyma appearing. There was no abnormal vascularity or calcification in the thyroid gland. There was no significant cervical lymphadenopathy.

A recent DEXA scan demonstrated a fairly stable bone mineral density of the spine and hip. However, measurement at the neck of the femur was particularly low at -3.5. This placed her in the imminent fracture risk group, which recommended the use of anabolic bone-protecting drugs. Romosozumab was recommended to be initiated to stop further use of hormone replacement therapy and to continue vitamin D supplement use.

The abdominal ultrasound scan showed no kidney stones. The thyroid function test was normal. On examination, she had no palpable neck mass. The endocrinology team referred her to the Ear, Nose, and Throat surgical team for consideration of parathyroidectomy.

**Figure 5:**
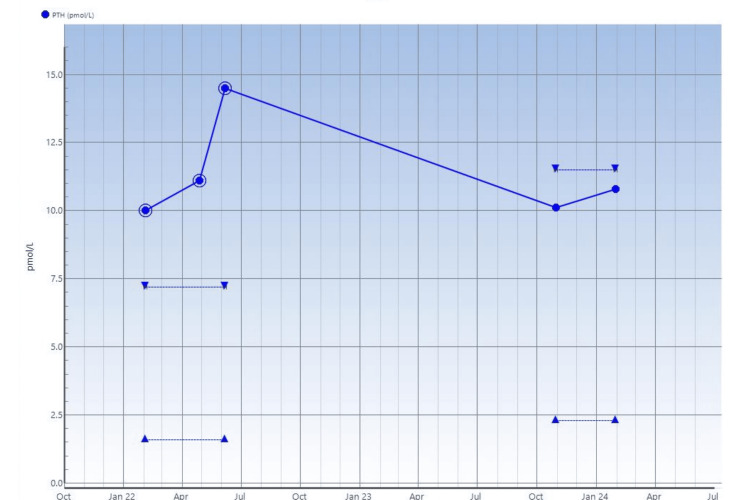
Serial serum PTH measurements on the patient Reference range: 2.3-11.5 pmol/l. One measurement of raised PTH levels noted, but the rest of the studies were normal. PTH: Parathyroid hormone

**Figure 6:**
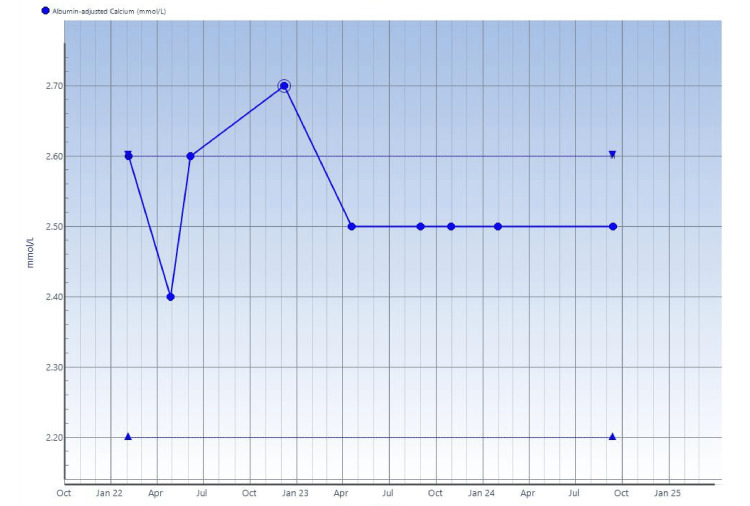
Serial serum albumin-adjusted total calcium measurements Periods of high measurements earlier noted, which eventually settled to normal limits.

**Figure 7:**
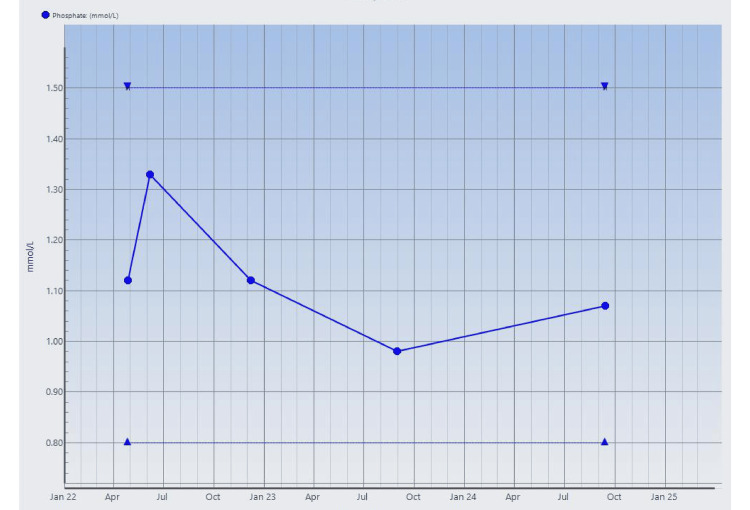
Serial serum phosphate measurements

**Figure 8:**
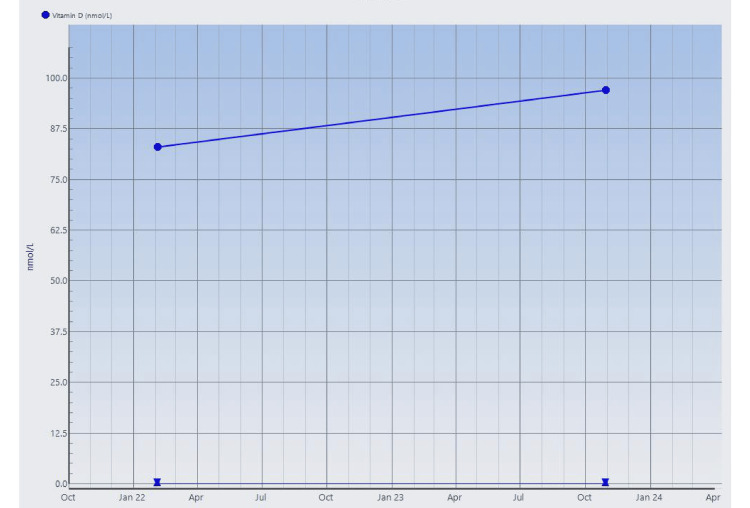
Serial serum vitamin D levels measured

Her recent blood test studies revealed normal serum calcium, phosphate, and vitamin D levels and normal PTH levels (Table 2, Figure 5-8).

**Table 2:** Results of latest laboratory studies on patient revealing normal serum parathormone, vitamin D, serum calcium and phosphate levels Fractional excretion of calcium (FECa) was calculated, and the result obtained was 1, suggesting a low likelihood of familial hypocalciuric hypercalcemia in this patient.

Test	Result	Reference range	Comments
Serum creatinine	49 umol/l	49-97 umol/l	Normal
Estimated glomerular filtration rate	98 ml/min	90-125ml/min	Normal study
Alkaline phosphatase	63 IU/l	44-147 IU/l	Normal study
Serum total calcium	2.6 mmo/l	2.2-2.6 mmol/l	At upper limit of normal reference range
Albumin	46 g/l	35-50 g/l	Normal study
Random urine calcium	1.8 mmol/l	1.25-3.75 mmol/l	Normal study
Urine creatinine	6 mmol/l	3.5-24.6 mmol/l	Normal study
Urine calcium	2.6 mmol/l	2.2-2.6 mmol/l	At upper limit of normal reference range
Urine creatinine	49 umol/l	44-80 umol/l	Normal study
Urine calcium: Creatinine ratio	0.0057	Less than 0.14	Normal study
Serum Vitamin D level	97 nmol/l	Greater than 50 nmol/l	Normal study
Serum parathyroid hormone (PTH) level	10.8 pmol/l	2.3-11.5 pmol/l	Normal study
Serum globulin	24 g/l	20-35 g/l	Normal study
IgG	8.4 g/l	6-16 g/l	Normal study
IgA	1.6 g/l	0.8-4.0 g/l	Normal study
IgM	1.9 g/l	0.5-2 g/l	Normal study
Serum electrophoresis	No paraprotein was detected.		
9 am plasma cortisol level	207 nmol/l	< 450nmol/l	Normal study

## Discussion

We present a rare finding of severe osteoporosis diagnosed at age 58 in a 65-year-old female who has a history of multiple fractures. Radiologic assessments indicated a potential parathyroid adenoma, yet her serum calcium, vitamin D, and parathyroid hormone levels were largely normal. Additionally, the patient underwent genetic screening, which returned negative results for familial hypocalciuric hypercalcemia and familial hyperparathyroidism.

Typically, the diagnosis of severe hyperparathyroidism-along with the identification of a parathyroid adenoma-arises during the evaluation of severe osteoporosis rather than as a result of symptomatic hypercalcemia [4]. In this particular case, however, we observe a rare juxtaposition: the presence of severe osteoporosis and parathyroid adenoma coexists with normal serum calcium, parathyroid hormone, and vitamin D levels. The patient also exhibited normal renal function upon evaluation. Instances in the literature that describe a similar scenario are exceptionally uncommon. This led the authors to hypothesize the possibility that parathyroid adenomas might produce other parathyroid hormone-like substances, which could exert a significant osteoclastic effect on bone, resulting in severe osteoporosis and multiple fractures.

A few instances of parathyroid adenoma associated with the production of parathyroid hormone-related peptide (PTHrP) have been documented in the literature. Gurrado A et al. suggested that PTHrP should not be exclusively considered in cases of malignancy [5]. The effect of PTHrP typically leads to sustained hypercalcemia, which is not present in the patient under review. Normally, PTHrP would suppress PTH levels, or those levels would remain within the normal range. Therefore, testing serum PTHrP could be warranted in the current case despite the low clinical suspicion for any underlying malignancy other than the radiologically identified parathyroid adenoma. Additionally, a completion positron emission tomography (PET) CT scan of the whole body or a CT scan of the chest, abdomen, and pelvis may be appropriate to rule out any other solid organ malignancies. 

Lafferty FW et al. (2006), in a publication discussing a 57-year-old male, proposed that possible post-translational modifications of PTH in the context of parathyroid adenoma might result in a misleading appearance of low PTH levels in conventional measurements. They noted that a greater number of PTH molecules were identified as serum dilution increased and recommended adjustments for dilution factors in similar scenarios. Further, they reported no intragenic mutations in the pre-pro-PTH coding region in either the parathyroid adenoma or matched blood DNA samples from the patient they studied [6].

Benaderet AD et al. (2011) reported a rare case involving a 14-year-old female diagnosed with primary hyperparathyroidism who presented with symptomatic hypercalcemia and a low serum PTH assay. Their findings highlighted discord between pre-operative serum PTH levels and intra-operative turbo PTH assay measurements. Notably, genomic analysis revealed no alterations in the PTH gene sequence, suggesting the potential occurrence of post-translational modifications to the PTH molecule. They posited that in clinical contexts where hyperparathyroidism is suspected yet characterized by low or normal PTH measurements, it is prudent to employ a variety of assays to evaluate PTH levels more comprehensively [7]. Although our reported case differs in that we observed normocalcemia and a normal PTH level, the recommendation to conduct a multifaceted assessment of PTH remains pertinent. 

Similarly, Bhadada SK et al. (2008) advocated for further investigation into the diverse intact PTH molecular species secreted by parathyroid adenomas and the post-translational modifications that could affect these molecules and hinder their in vitro quantification. They emphasized the need to explore the underlying mechanisms contributing to these phenomena [8].

The World Health Organization's criteria for the interpretation of bone mineral density (BMD) classify individuals into three categories: "Normal" (T-score at or above -1.0), "Osteopenic" (T-score between -1.0 and -2.5), and "Osteoporotic" (T-score at or below -2.5) [9]. In the case under consideration, the patient was classified as osteoporotic and had significant management challenges. Ultimately, the treatment protocol included the administration of romosozumab, as teriparatide was deemed contraindicated due to the presence of a parathyroid adenoma. Gupta V et al. posit that a lack of response to teriparatide in patients with severe osteoporosis may indicate underlying conditions such as hypocalcemic hyperparathyroidism or other secondary causes of osteoporosis. They advocate for comprehensive metabolic evaluations, including routine assessment of parathyroid hormone levels prior to initiating teriparatide therapy [10]. In the case examined by Gupta et al., the 61-year-old female subject demonstrated notably elevated pre-operative parathyroid hormone levels, which contrasted with the patient's profile in our current study. Additionally, Lowe H et al. also observed that individuals with normocalcemic hyperparathyroidism in a single-center, follow-up observational study exhibited more extensive skeletal involvement compared to those with classic hypercalcaemic primary hyperparathyroidism. They posited that normocalcaemic hyperparathyroidism may represent an earlier form of symptomatic primary hyperparathyroidism as opposed to being an asymptomatic form of primary hyperparathyroidism [11]. The suggestion that the possible resistance to parathormone in the skeletal system of patients with normocalcaemic hyperparathyroidism could account for the severe disease manifestation was shared by Leslie SW et al. (2024) [3]. 

Romosozumab is a monoclonal antibody approved for the management of severe postmenopausal osteoporosis in patients identified as having a significant risk of fracture. This medication is administered via monthly injections over a 12-month course, with the expectation that patients will be trained to self-administer the injections. The primary side effects associated with romosozumab include joint pain and headaches. Additionally, there is a rare risk of cardiac events, necessitating baseline assessments before the initiation of treatment. Another rare but noteworthy side effect is osteonecrosis of the jaw, which requires that patients be advised to cease treatment immediately should they encounter any dental issues [12]. While romosozumab is known to enhance bone mineral density, it has also been noted to be able to lower serum calcium levels while concurrently increasing PTH levels [13].

## Conclusions

Further research is needed to explore the rare occurrence of parathyroid adenoma in patients with normocalcemia, normal parathyroid hormone (PTH) levels, normal vitamin D levels, and normal renal function tests. The potential for a delay in the radiological diagnosis of parathyroid adenoma and onward definitive surgical intervention could result where initial biochemical parameters from studies done for parathyroid adenoma fall within normal reference limits. Clinicians should be aware of such atypical findings. The release of PTHrP by certain parathyroid adenomas is beginning to be recognized in the literature. Typically, PTHrP is associated with hypercalcemia and low or suppressed PTH levels. In some cases of parathyroid adenoma, post-translational changes may lead to the production of specific PTH levels that standard serum PTH assays may not detect. Some studies have suggested increasing serum dilution during PTH sample analysis and adjusting for dilution factors in cases where parathyroid adenoma is present, but regular assays yield low serum PTH levels.
